# Comprehensive geriatric assessment in patients undergoing transcatheter aortic valve implantation – results from the CGA-TAVI multicentre registry

**DOI:** 10.1186/s12872-017-0740-x

**Published:** 2018-01-04

**Authors:** Andrea Ungar, Giulio Mannarino, Nathalie van der Velde, Jan Baan, Marie-Pierre Thibodeau, Jean-Bernard Masson, Gennaro Santoro, Martijn van Mourik, Sofie Jansen, Cornelia Deutsch, Peter Bramlage, Jana Kurucova, Martin Thoenes, Stefania Maggi, Andreas W. Schoenenberger

**Affiliations:** 1 Geriatric Intensive Care Unit, Department of Geriatrics and Medicine, Careggi Hospital and University of Florence, Florence, Italy; 2 Internal Medicine, Section of Geriatric Medicine, Academic Medical Center, Amsterdam, Netherlands; 3 Cardiology, Academic Medical Center, Amsterdam, Netherlands; 4 Centre Hospitalier de l’Université de Montréal, Montréal, Canada; 5 Institute for Pharmacology und Preventive Medicine, Cloppenburg, Germany; 6 Edwards Lifesciences, Nyon, Switzerland; 7 CNR–Institute of Neuroscience, Aging Branch, Padua, Italy; 8 Department of Geriatrics, Inselspital, Bern University Hospital, University of Bern, Bern, Switzerland

**Keywords:** Transcatheter aortic valve implantation (TAVI), Comprehensive geriatric assessment (CGA), Multidimensional prognostic index (MPI), Short physical performance battery (SPPB), Silver code

## Abstract

**Background:**

In older patients with aortic stenosis (AS) undergoing TAVI, the potential role of prior CGA is not well established. To explore the value of comprehensive geriatric assessment (CGA) for predicting mortality and/or hospitalisation within the first 3 months after transcatheter aortic valve implantation (TAVI).

**Methods:**

An international, multi-centre, prospective registry (CGA-TAVI) was established to gather data on CGA results and medium-term outcomes in geriatric patients undergoing TAVI. Logistic regression was used to evaluate the predictive value of a multidimensional prognostic index (MPI); a short physical performance battery (SPPB); and the Silver Code, which was based on administrative data, for predicting death and/or hospitalisation in the first 3 months after TAVI (primary endpoint).

**Results:**

A total of 71 TAVI patients (mean age 85.4 years; mean log EuroSCORE I 22.5%) were enrolled. Device success according to VARC criteria was 100%. After adjustment for selected baseline characteristics, a higher (poorer) MPI score (OR: 3.34; 95% CI: 1.39–8.02; *p* = 0.0068) and a lower (poorer) SPPB score (OR: 1.15; 95% CI: 1.01–1.54; *p* = 0.0380) were found to be associated with an increased likelihood of the primary endpoint. The Silver Code did not show any predictive ability in this population.

**Conclusions:**

Several aspects of the CGA have shown promise for being of use to physicians when predicting TAVI outcomes. While the MPI may be useful in clinical practice, the SPPB may be of particular value, being simple and quick to perform. Validation of these findings in a larger sample is warranted.

**Trial registration:**

The trial was registered in ClinicalTrials.gov on November 7, 2013 (NCT01991444).

## Background

Severe symptomatic stenosis of the aortic valve (aortic stenosis; AS) is associated with mortality of up to 50% at 1 year if left untreated [1, 2]. The outcome of surgical aortic valve replacement (SAVR) is generally predicted with the aid of quantitative scales such as the EuroSCORE or the Society of Thoracic Surgeons (STS) risk score. However, the accuracy of such algorithms for assessing older-age, multi-morbid patients undergoing transcatheter aortic valve implantation (TAVI) is low [3–5]. This is mainly due to the absence of variables related to ageing, such as frailty, mental status, social support, and overall health. There is evidence that additionally evaluating these factors can help provide a more precise estimation of an older person’s response to treatment [3, 6, 7]. Indeed, a recent report from the American College of Cardiology (ACC) advocates assessment of frailty and cognitive function prior to determining a patient’s suitability for TAVI [8]. The inclusion of a geriatrician in the Heart Team responsible for assessing patients prior to TAVI may therefore be warranted [9].

A comprehensive geriatric assessment (CGA) is a multidimensional diagnostic process for evaluating an individual’s clinical, psychosocial, and functional characteristics [10, 11]. It usually consists of functional tests complementing usual clinical evaluation. For example, CGA may include the calculation of a multidimensional prognostic index (MPI) based on mental and nutritional status, number of co-morbid conditions and medications, living arrangements, and the ability to cope with activities of daily living [12, 13]. Pilotto et al. showed that the MPI had high predictive power for assessing mortality after hospitalisation of older patients [13]. For TAVI specifically, few studies have evaluated CGA and its components for outcome prediction [3, 14–16]. For example, Stortecky et al. evaluated a geriatric assessment that contained many of the same components as the MPI, and found that many of the included items were predictive of mortality and the occurrence of a major adverse cardiac or cerebrovascular event (MACCE) at 30 days and 1 year after the procedure [14]. All previous studies originated from single-centre experiences.

We aimed to determine the power of CGA for predicting the combined endpoint of mortality and stroke within the 3 months subsequent to TAVI based on data from a multi-centre, prospective cohort. We further characterised changes in CGA over time, and provide additional evidence for the utility of TAVI in a geriatric, comorbid population.

## Methods

### Patients and registry design

The CGA-TAVI registry is a prospective, international, multi-centre, observational registry [17]. Patients were enrolled at three centres in Italy (Careggi Hospital, Florence), the Netherlands (Academic Medical Center, Amsterdam) and Canada (Centre Hospitalier de l’Université de Montréal) between August 2013 and December 2015. Individuals were eligible if they were aged >80 years, had symptomatic severe calcific aortic valve (AV) stenosis, and were assigned to undergo transaortic, transapical or transfemoral TAVI. Patients were excluded if TAVI was being performed as an emergency procedure or if patients were unable to participate in the follow-up. Overall, 603 patients underwent TAVI at one of the three study centres during the study period (Italy: 68, Canada: 95; the Netherlands: 440. Of these, 71 patients were enrolled in the CGA-TAVI registry: 41, 15 and 15 from the sites in Italy, Canada and the Netherlands, respectively.

### Baseline assessment

A detailed description of the information documented has been previously published [17]. Briefly, data regarding demographics, comorbidities, and prior cardiovascular interventions were collected at hospital admission. A CGA was performed by a geriatrician for each patient. This included calculation of the MPI [12, 13], which consisted of the following components: Activities of Daily Living (ADL; 6 items) [18, 19]; Instrumental Activities of Daily Living (IADL; 8 items) [20]; Short Portable Mental Status Questionnaire (SPMSQ; 10 items) [21]; Cumulative Illness Rating Scale (CIRS; 14 items) [22, 23]; Mini Nutritional Assessment (MNA; 18 items) [24]; Exton-Smith Scale (ESS; 5 items) [25]; number of drugs used (1 item); and co-habitation status (1 item). In each case, a tripartite hierarchy was used for scoring (no problems: 0 points; minor problems: 0.5 points; severe problems: 1 point). The boundaries for these scores were based on the cut-off points derived from the associated literature [12]. A Silver Code value was also calculated from administrative data for further prognostic stratification [26] and a Short Physical Performance Battery (SPPB) was performed, which involved repeated chair stands, balance testing, and an 8-ft (2.44 m) walk [27]. Detailed breakdowns of the three assessment scores can be found in Appendices 1, 2 (MPI), 3 (Silver Code) and 4 (SPPB). Procedural characteristics were also documented.

### Follow-up assessment and outcomes

Patients were scheduled for follow-up at discharge, 30 days and 3 months post-procedure. These visits were conducted at the patient’s enrolling centre and involved repetition of the CGA performed at the baseline assessment. Death, all-cause hospitalisation, TAVI-related hospitalisation, stroke, transient ischaemic attack (TIA), myocardial infarction (MI), life-threatening bleeding, acute kidney injury, coronary artery obstruction requiring intervention, major vascular complications, valve dysfunction requiring repeat procedure, or worsening congestive heart failure (CHF) were recorded, as defined in the Valve Academic Research Consortium-2 (VARC-2) consensus document [28].

### Endpoints

The primary endpoint was death and/or hospitalisation within the first 3 months after TAVI. The secondary endpoint was death and/or non-fatal stroke within the same period. Changes in the scores of the components of the CGA from baseline to 3 months were also evaluated.

### Data management and statistics

Data were entered into an online database via the completion of an electronic case report form (eCRF; s4trials, Berlin, Germany). Details were automatically checked for plausibility and completeness.

Data were analysed using descriptive statistics, with categorical variables presented as absolute values and percentages and continuous variables as means with standard deviations (SD). A logistic regression was used to evaluate the predictive value of CGA components (MPI, SPBB and Silver Code) for the primary/secondary endpoints. Age, gender, New York Heart Association (NYHA) class and surgical risk (EuroSCORE/STS) were used as co-variables. Logistic regression results are presented as odds ratios (OR) with 95% Wald confidence limits (95% CI) and *p*-values. CGA changes from baseline to 3-month follow-up were tested for significance using a t-test. *P*-values of <0.05 were considered significant.

Data were analysed using IBM SPSS statistics version 24 (IBM corporation, Amonk, New York, USA).

## Results

### Baseline patient characteristics

Overall, 44 patients (62%) were female. Means for age and body mass index (BMI) were 85.4 ± 2.9 years and 24.7 ± 3.7 kg/m^2^, respectively (Table 1). The most prevalent comorbidity was hypertension (83.1%), followed by coronary artery disease (53.5%), peripheral artery disease (28.2%), diabetes mellitus (26.8%), prior MI (23.9%), and pulmonary disease (15.5%). In terms of surgical risk, the mean log EuroSCORE and STS scores were 22.5 ± 13.2% and 5.8 ± 3.9%, respectively.

**Table 1:** Patient characteristics

	Mean ± SD (n) / n/N (%)
Age [years]	85.4 ± 2.9 (*n* = 71)
Gender [female]	44/71 (62.0)
BMI [kg/m^2^]	24.7 ± 3.7 (*n* = 71)
Comorbidities	
Hypertension	59/71 (83.1)
Diabetes mellitus	19/71 (26.8)
PAD	20/71 (28.2)
Prior stroke/TIA	6/71 (8.5)
CAD	38/71 (53.5)
Prior MI	17/71 (23.9)
Pulmonary disease^a^	11/71 (15.5)
Pulmonary hypertension	35/71 (49.3)
Creatinine ≥2.0 mg/dl^b^	5/71 (7.0)
Dialysis	2/71 (2.8)
Prior cardiovascular intervention	
PCI	16/71 (22.5)
CABG	13/71 (18.3)
Mitral valve replacement	2/71 (2.8)
Tricuspid valve replacement	0/71 (0)
Balloon aortic valvuloplasty	6/71 (8.5)
PPI	5/71 (7.0)
Surgical risk	
Log EuroSCORE I	22.5 ± 13.2 (*n* = 71)
STS risk score	5.8 ± 3.9 (*n* = 71)
AS-related symptoms (%)	
Syncope	5/71 (7.0)
Dizziness with exertion	5/71 (7.0)
CCS angina grade	
Class III	7/70 (10.0)
Class IV	0/70 (0)
NYHA classification	
Class III	50/71 (70.4)
Class IV	7/71 (9.9)
AS echocardiographic parameters	
AV peak gradient (mmHg)	78.5 ± 17.8 (*n* = 59)
AV mean gradient (mmHg)	50.5 ± 14.1 (*n* = 61)
V_max_ (m/s)	4.2 ± 0.9 (*n* = 24)
Effective orifice area (cm^2^)	0.9 ± 0.6 (*n* = 37)
LVEF (%)	50.9 ± 12.0 (*n* = 62)

Legend: *BMI* body mass index, *PAD* peripheral artery disease, *TIA* transient ischaemic attack, *CAD* coronary artery disease, *MI* myocardial infarction, *PCI* percutaneous coronary intervention, *CABG* coronary artery bypass grafting, *PPI* permanent pacemaker implantation, *log Euro SCORE* logistic European System for Cardiac Operative Risk Evaluation, *STS* Society of Thoracic Surgeons, *AS* aortic stenosis, *LVEF* left ventricular ejection fraction, *AV* aortic valve, *CCS* Canadian Cardiovascular Society, *NYHA* New York Heart Association, *V*_*max*_ maximum velocity

^a^Defined as chronic obstructive pulmonary disease, asthma, a forced expiratory volume-1 of < 1.0, or oxygen dependency

^b^Excluding patients with dialysis

The majority of patients were at NYHA class III (70.4%) or IV (9.9%). Further AS-related symptoms were class III angina (10.0%), dizziness with exertion (7.0%), and syncope (7.0%). Regarding echocardiography, peak and mean AV gradients were 78.5 ± 17.8 and 50.5 ± 14.1 mmHg, respectively, with a left ventricular ejection fraction (LVEF) of 50.9 ± 12.0%, a mean V_max_ of 4.2 ± 0.9 m/s, and an effective orifice area of 0.9 ± 0.6 cm^2^.

### Procedural characteristics and periprocedural outcomes

Details about procedural aspects are provided in the supplementary data (Appendix 5). Devices were placed successfully (as defined by VARC-2 [28]) in all patients (Appendix 6). The rate of intraoperative complications was 17.1%, which were vascular complications requiring treatment in 10.0% of patients and access-related in 5.6%. No conversion to open surgery was necessary. Paravalvular regurgitation was moderate in 2.9%, with no severe regurgitation. Post-procedural AV peak and mean gradients were 20.0 ± 12.4 and 11.3 ± 7.2 mmHg, respectively.

### Outcomes at 3-months

In total, 6 patients died (8.5%) and 9 were hospitalised (13.8%) by the 3-month follow-up. The primary endpoint (death and/or hospitalisation in the first 3 months) was observed in 13/71 patients (18.3%) (Table 2). After adjustment for baseline characteristics, a higher (poorer) MPI score (OR: 3.34; 95% CI: 1.39–8.02; *p* = 0.0068) and a lower (poorer) SPPB score (OR: 1.15; 95% CI: 1.01–1.54; *p* = 0.0380) were found to be associated with an increased odds of primary endpoint achievement (Table 3).

**Table 2:** Short- and medium-term outcomes

	≤30 days^a^ n/N (%)	≤3 months n/N (%)
Primary endpoint (Death and/or hospitalisation)	5/70 (7.1)	13/71 (18.3)
Secondary endpoint (Death and/or non-fatal stroke)	2/71 (2.8)	6/71 (8.5)
All-cause mortality	2/71 (2.8)	6/71 (8.5)
Non-fatal complications	(*n* = 69)	(*n* = 65)
All-cause hospitalisation	5/69 (7.2)	9/65 (13.8)
Valve-related hospitalisation	3/69 (4.3)	2/65 (6.2)
Non-fatal stroke	0/69 (0.0)	0/62 (0.0)
Acute kidney injury (stage 2 or 3)	3/69 (4.3)	5/64 (7.8)
Major vascular complication	1/69 (1.4)	0/64 (3.1)
Repeat procedure for valve dysfunction	0/69 (0)	1/62 (1.6)
Myocardial infarction	0/69 (0)	0/62 (0)
PPI	9/69 (13.0)	9/65 (13.8)

Legend: *CHF* congestive heart failure, *MI* myocardial infarction, *NYHA* New York Heart Association, *CCS* Canadian Cardiovascular Society, *PPI* permanent pacemaker implantation

^a^≤ 30 days includes all complications which occurred periprocedurally, during the phase of hospitalisation for TAVI and after discharge within 30 days

**Table 3:** Logistic regression for the prediction of events at 3 months by CGA at baseline

	Univariable OR (95% CI)	*p*-value	Multivariable OR (95% CI)	*p*-value
Death and/or hospitalisation				
Increasing MPI score (high vs. low)	0.66 (0.54–0.81)	< 0.0001	3.34 (1.39–8.02)^a^	0.0068
Decreasing SPPB (low vs. high)	1.35 (1.19–1.53)	< 0.0001	1.15 (1.01–1.54)	0.0380
Increasing Silver Code (high vs. low)	0.94 (0.92–0.97)	< 0.0001	1.03 (0.91–1.15)	0.6576
Death and/or non-fatal stroke				
Increasing MPI score (high vs. low)	0.49 (0.36–0.63)	< 0.0001	4.75 (1.40–16.08)	0.0123
Decreasing SPPB (low vs. high)	1.89 (1.36–2.64)	0.0002	1.62 (1.08–2.43)	0.0188
Increasing Silver Code (high vs. low)	0.90 (0.87–0.94)	< 0.0001	1.04 (0.87–1.23)	0.6938

Legend: *MPI* multidimensional prognostic index, *SPPB* short physical performance battery. All values adjusted for age, gender, NYHA class and surgical risk (EuroSCORE)

^a^The direction of the OR changed with the introduction of age into the model

The secondary endpoint (death and/or non-fatal stroke in the first 3 months) was observed in 6 patients (8.5%) (Table 2). After multivariate adjustment, a higher (poorer) MPI score (OR: 4.75; 95% CI: 1.40–16.08; *p* = 0.0123) and a lower (poorer) SPPB score (OR: 1.62; 95% CI: 1.08–2.43; *p* = 0.0188) were associated with a greater odds of secondary endpoint achievement (Table 3).

### CGA change from baseline to 3 months

Between baseline and 3 months, the total MPI score decreased only slightly, from a mean of 0.34 ± 0.11 to 0.30 ± 0.13 (mean intra-individual change: −0.02 ± 0.12; *p* = 0.25) (Appendix 7). However, the CIRS and ESS components changed by a statistically significant amount (−0.12 ± 0.25; *p* < 0.001 and −0.04 ± 0.13; *p* = 0.04, respectively). While the change in Silver Code was small, the SPPB score increased significantly (+1.86 ± 2.76; p < 0.001).

## Discussion

Of the multiple components of the CGA that were evaluated, the MPI and the SPPB both had value for predicting the likelihood of death and/or hospitalisation in the first 3 months following TAVI. In terms of time-efficiency, the SPPB appears to be the favourable approach, with the MPI perhaps not adding sufficient additional predictive value to warrant such a time-consuming assessment.

### Outcomes

In our patients, the rate of all-cause mortality at 30 days (2.8%) was within the range (1.1%–5.9%) reported by large-scale studies in patients with a similar level of surgical risk and mean ages above 80 years, such as the PARTNER II trial and SOURCE 3, WIN-TAVI, Swiss TAVI, and PRAGMATIC registries [29–33]. Though a less commonly reported outcome, the same was true of the rate of rehospitalisation (7.2% vs. 4.6% in the PARTNER II SAPIEN 3 trial and 6.5% in the overall PARTNER II trial) [29, 34]. Variations in rates between studies are likely due to differing patient characteristics, access routes, and the valves/delivery devices available during study periods. Our findings regarding three-month outcomes could not be easily compared to previous studies, as this time-point is not largely reported upon in the literature.

### Surgical risk scores

EuroSCORE and STS algorithms are conventional tools for assessing cardiac operative risk. It has been suggested that the latter is slightly more accurate in TAVI patients [4, 5], though neither are ideal. Patients in the present study had expected mortality rates of 22.5% (EuroSCORE I) and 5.8% (STS), and though the latter was closer to the observed rate, both were excessively elevated. Even the more up-to-date EuroSCORE II has been shown to have suboptimal discriminatory power, suggesting the need for alternative or additional assessment tools [35, 36].

### Multidimensional prognostic index

Use of a CGA during clinical assessment of operative risk in AS patients has been suggested as a way to address the shortcomings of the EuroSCORE and STS score, and better predict outcomes [17, 36]. In the present study, both MPI and SPPB were found to have predictive value for determining the likelihood of short-term mortality/hospitalisation or stroke after TAVI. Interestingly, while high MPI was a “negative predictor” in the univariate analysis, it became a “positive predictor” after adjusting for age, gender, NYHA class and surgical risk. This change of direction can be explained by the clinical setting; younger patients with a low MPI are typically treated with SAVR, while elderly patients with a high MPI are unlikely to be treated at all. Consequently, our TAVI population was likely composed of patients with a lower age and high MPI or a higher age and low MPI, resulting in a switch of the direction of the odds ratio at multivariate analysis. This reflection of the clinical context is supportive of the “real” association between MPI score and outcomes.

Though no other studies appear to have specifically reported on the predictive value of MPI in TAVI, several have shown higher MPI scores to be significantly associated with greater rates of mortality in older patients with a variety of acute illnesses, including heart failure and TIA [12, 13, 37–40]. Other studies have evaluated other multi-component models for predicting mortality and morbidity after cardiac surgery [41]. For TAVI specifically, Green et al. found that patients with a high frailty score, as determined by gait speed, grip strength, serum albumin and ADL, were at greater risk of one-year mortality [15]. The five-component frailty score proposed by Kamga et al. was found to predict one-year mortality after transfemoral TAVI [16]. Stortecky et al. identified numerous parameters in their Multidimensional Geriatric Assessment that were predictive of 30-day and one-year mortality and MACCE after TAVI [14]. Data from the PARTNER trial were used to construct models for predicting a poor outcome, defined as death or a low/significantly decreased quality of life, after TAVI [42]. These models were subsequently validated in a large multi-centre cohort of TAVI patients, with an incremental increase in discriminative ability identified on the addition of markers of frailty and disability [43]. In agreement with the data from our CGA registry, these studies demonstrate the potential value of such multi-component analyses for predicting outcome after TAVI.

### Short physical performance battery

A significant drawback of these multidimensional evaluation tools, however, is that they are extremely time-consuming. In the present work, we found that use of the SPPB alone was equally as effective as the MPI for predicting death and/or hospitalisation, and death and/or non-fatal stroke, in the first 3 months after TAVI. This short series of tests is recommended by the European Union Geriatric Medicine Society (EUGMS) as part of a CGA in older AS patients [17], although it appears that there is little published evidence in support of using it for assessing TAVI candidates specifically. The concept of tests of physical ability to predict outcome after cardiac surgery has been evaluated in other studies. Afilalo et al. demonstrated that a slow 5-m gait speed was associated with a greater risk of operative mortality and in-hospital mortality and major morbidity in older patients undergoing cardiac surgery [7, 44]. They further determined that use of this parameter alone was superior to a variety of other frailty scales [6]. In patients undergoing TAVI specifically, 5-m gait speed has been shown to be independently associated with 30-day mortality after adjustment for STS score and other relevant baseline characteristics [45]. Stortecky et al. reported that the “timed get-up and go” (TUG) test had the greatest predictive ability of all of the individual geriatric assessment tools that they investigated [14]. In combination with either the STS or EuroSCORE, the TUG was superior to the other components evaluated for predicting all-cause mortality and MACCE during the first year after TAVI. A recent report by the ACC recommends that a 5-m gait speed test and a 6 min walk test be used to assess frailty and physical functioning, respectively, when determining a patient’s suitability for TAVI [8].

The simplicity of physical tests such as the SPPB is not their only advantage, with the lack of subjectivity on the part of both physician and patient providing a level of accuracy that cannot be obtained using questionnaire-based assessment. This is particularly relevant for the advanced-age TAVI population, where cognitive impairment is a potentially significant confounding factor when evaluating self-reported parameters [6].

### Silver code

According to our data, the Silver Code had no value for predicting death/hospitalisation or death/stroke during the first 3 months after TAVI. The calculation of this parameter prior to deciding on the suitability of a patient for TAVI is another recommendation of the EUGMS [17]. Previous studies have demonstrated a relationship between Silver Code and one-year mortality, although this was in the setting of the Emergency Department [26, 46]. As the Silver Code is determined from administrative data, it is particularly suited to planned procedures such as TAVI. Therefore, although it was not found to be an independent predictor of outcome in the present analysis, it should perhaps not be discounted. Further evaluation in a larger population may clarify its utility as part of a CGA prior to TAVI.

### Limitations

Firstly, as an observational study, inherent limitations such as a higher potential for missing data are present. However, the observational aspect carried several advantages, such as an evaluation of TAVI patients in a real-world setting, avoiding the confounding issue of the strict inclusion and exclusion criteria used for clinical trials. This is particularly important, as geriatric patients are often those excluded due to high levels of comorbidity. Secondly, our findings are currently only applicable to geriatric patients at higher surgical risk. Considering that there is a current shift in clinical practice towards TAVI in lower-risk patients who are normally eligible for surgical heart valve replacement, re-evaluation of CGA in different populations may become necessary. In addition, we were only able to obtain data for a modest number of patients from three participating sites, limiting statistical power and generalisability. Indeed, the relatively low incidence of mortality and stroke at 3 months in a fairly small sample may have resulted in suboptimal power to detect baseline characteristics that are predictive for this outcome. Future studies in larger samples would be useful for clarification.

## Conclusion

Several aspects of the CGA have shown promise for being of use to physicians when predicting the likelihood of death, rehospitalisation and non-fatal stroke following TAVI. The strong association between MPI and such outcomes indicates its potential utility in clinical practice. In addition, the SPPB may have significant value, being simple and quick to perform; however, the modest sample size included herein limit the formation of firm conclusions, and validation of these findings in a larger sample of TAVI patients with a greater range of surgical risk is warranted.

## Clinical perspectives

The accuracy of conventional surgical risk scores is known to be suboptimal for predicting the outcomes of TAVI in elderly aortic stenosis patients. This study shows a potential benefit of adding items from a comprehensive geriatric assessment (CGA) to pre-intervention assessments. This now requires further validation in larger cohorts.

## Appendix 1

Provides an overview about the Multidimensional Prognostic Index (MPI).

**Table 4:** Calculation of the Multidimensional Prognostic Index (MPI)

	Problem severity		
	No (= 0 points)	Minor (= 0.5 points)	Severe (= 1 point)
1. Co-habitation status	Living with relatives/nurse	Living in an institution	Living alone
2. Current medication use	0–3 medications	4–6 medications	≥7 medications
3. ADL score	6–5	4–3	2–0
4- IADL score	8–6	5–4	3–0
5. SPMSQ score	0–3	4–7	8–10
6. ESS score	16–20	10–15	5–9
7. CIRS CI	0	1–2	≥3
8. MNA score	≥24	17–23.5	<17
Total MPI score (sum of points/8):			
Low-risk:	≤0.33		
Moderate-risk:	0.34–0.66		
High-risk:	>0.66		

Legend: *ADL* activities of daily living, *IADL* instrumental ADL, *SPMSQ* short portable mental status questionnaire, *ESS* Exton-Smith scale, *CIRS* cumulative illness raiting scale, *CI* comorbidity index, *MNA* mini nutritional assessment, *MPI* multidimensional prognostic index. The numbering on the left-hand side of factors corresponds to the numbering in Appendix 2

## Appendix 2

Provides a breakdown of the Multidimensional Prognostic Index (MPI).

**Table 5:** Breakdown of MPI Scoring

3. Activities of Daily Living (ADL)	Points				
Bathing (sponge bath, tub bath, or shower)					
Receives no assistance (gets in and out of the tub by self if tub is usual means of bathing)	1				
Receives no assistance in bathing only one part of the body (such as back or leg)	1				
Receives assistance in bathing more than one part of the body (or not bathed)	0				
Dressing (gets clothes from closets and drawers, including underclothes/outer garments and using fasteners/braces, if worn)					
Gets clothes and gets completely dressed without assistance	1				
Gets clothes and gets dressed without assistance except for assistance in tying shoes	1				
Receives assistance in getting clothes or in getting dressed, or stays partly or completely undressed	0				
Toileting					
Goes to “toilet room” cleans self, and arranges clothes without assistance (may use object for support such as cane, walker, or wheelchair and may manage night bedpan or commode, emptying same in morning)	1				
Receives assistance in going to “toilet room” or in cleaning self or in arranging clothes after elimination or in use of night bedpan or commode	0				
Doesn’t go to room termed “toilet” for the elimination process	0				
Transfer					
Moves in and out of bed as well as in and out of chair without assistance (may be using object for support such as cane or walker)	1				
Moves in and out of bed or chair with assistance	0				
Doesn’t get out of bed	0				
Continence					
Controls urination and bowel movement completely by self	1				
Has occasional “accidents”	0				
Supervision helps keep urine or bowel control, catheter is used, or is incontinent	0				
Feeding					
Feeds self without assistance	1				
Feeds self except for getting assistance in cutting meat or buttering bread	1				
Receives assistance in feeding or is fed partly or completely by using tubes or intravenous fluids	0				
Max ADL score (best performance):	6				
4. Instrumental Activities of Daily Living Scale (IADL)					
Ability to use telephone					
Operates telephone on own initiative: Iooks up and dials numbers, etc.	1				
Dials a few well-known numbers	1				
Answers telephone but does not dial	1				
Does not use telephone at all	0				
Shopping					
Takes care of all shopping needs independently	1				
Shops independently for small purchases	0				
Needs to be accompanied on any shopping trip	0				
Completely unable to shop	0				
Food preparation					
Plans, prepares and serves adequate meals independently	1				
Prepares adequate meals if supplied with ingredients	1				
Heats, serves and prepares meals or prepares meals but does not maintain adequate diet	0				
Needs to have meals prepared and served	0				
Housekeeping					
Maintains house alone or with occasional assistance (e.g. “heavy work domestic help”)	1				
Performs light daily tasks such as dishwashing, bed-making	1				
Performs light daily tasks but cannot maintain acceptable level of cleanliness	1				
Needs help with all home maintenance tasks	0				
Does not participate in any housekeeping tasks	0				
Laundry					
Does personal laundry completely	1				
Launders small items; rinses stockings, etc.	1				
All laundry must be done by others	0				
Mode of transportation					
Travels independently on public transportation or drives own car	1				
Arranges own travel via taxi, but does not otherwise use public transportation	1				
Travels on public transportation when accompanied by another	1				
Travel limited to taxi or automobile with assistance of another	0				
Does not travel at all	0				
Responsibility for own medications					
Is responsible for taking medication in correct dosages at correct time	1				
Takes responsibility if medication is prepared in advance in separate dosage	0				
Is not capable of dispensing own medication	0				
Ability to handle finances					
Manages financial matters independently (budgets, writes checks, pays rent, bills, goes to bank), collects and keeps track of income	1				
Manages day-to-day purchases, but needs help with banking, major purchases, etc	1				
Incapable if handling money	0				
Max IADL score (best performance):	8				
5. Short Portable Mental Status Questionnaire (SPMSQ)					
What is the date today? (Correct only when the month, date, and year are all correct)	If incorrect: 1				
What day of the week is it?	If incorrect: 1				
What is the name of this place? (Correct if any of the description of the location is given)	If incorrect: 1				
What is your street address?	If incorrect: 1				
How old are you?	If incorrect: 1				
When were you born?	If incorrect: 1				
Who is the president (or the Pope) now? (Requires only the correct last name)	If incorrect: 1				
Who was president (or the Pope) just before him?	If incorrect: 1				
What was your mother’s maiden name?	If incorrect: 1				
Subtract 3 from 20 and keep subtracting 3 from each new number at least for 3 times (the entire series must be performed correctly to be scored as correct)	If incorrect: 1				
Max SPMQ score (worst performance):	10				
6. Exton-Smith Scale (ESS)					
General Condition					
Bad	1				
Poor	2				
Fair	3				
Good	4				
Mental State					
Stuporous	1				
Confused	2				
Apathetic	3				
Alert	4				
Activity					
In bed all day	1				
Chairfast	2				
Walks with help	3				
Ambulant	4				
Incontinence					
Doubly incontinent	1				
Usually of urine	2				
Occasional	3				
None	4				
Mobility in Bed					
Immobile	1				
Very limited	2				
Slightly limited	3				
Full	4				
Max ESS score (best performance):	20				
7. Cumulative Illness Rating Scale					
	Point allocation based on disease severity				
	None	Mild	Moderate	Severe	Extremely severe
Cardiac (heart only)	1	2	3	4	5
Hypertension (rating is based on severity)	1	2	3	4	5
Vascular (arteries, veins, lymphatics)	1	2	3	4	5
Respiratory (lungs, bronchi, trachea)	1	2	3	4	5
EENT (eye, ear, nose, throat, Iarynx)	1	2	3	4	5
Upper GI (esophagus, stomach, duodenum, biliary and pancreatic trees)	1	2	3	4	5
Lower GI (intestines, hernias)	1	2	3	4	5
Hepatic (liver only)	1	2	3	4	5
Renal (kidneys only)	1	2	3	4	5
Other GU (urethers, bladder, urethra, prostate, genitals)	1	2	3	4	5
Musculo-skeletal-integumentary (muscles, bone, skin)	1	2	3	4	5
Neurological (brain, spinal cord, nerves)	1	2	3	4	5
Endocrine-metabolic (including diabetes, hyperlipidemia, infections, toxicity)	1	2	3	4	5
Psychiatric (dementia, depression, anxiety, agitation, psychosis)	1	2	3	4	5
Max comorbidity index (number of items with a score of ≥3; excluding the psychiatric item; most severe):	13				
8. Mini Nutritional Assessment (MNA)	Points				
Anthropometric Assessment					
Body Mass Index					
< 19	0				
19–20	1				
21–22	2				
≥ 23	3				
Mid-arm circumference (cm)					
< 21	0				
22	0.5				
> 22	1				
Calf circumference (cm)					
< 31	0				
> 31	1				
Weight loss (last 3 months)					
Loss of >3 kg	0				
Do not know	1				
Loss between 1 and 3 kg	2				
No weight loss	3				
General Assessment					
Lives independently (not in a nursing home or hospital)					
No	0				
Yes	1				
Takes more than 3 prescription drugs per day					
Yes	0				
No	1				
Has suffered psychological stress or acute disease in the past 3 months					
Yes	0				
No	1				
Mobility					
Bed/chair-bound	0				
Able to get out of bed / chair but does not go out	1				
Goes out	2				
Neuropsychological problems					
Severe dementia/depression	0				
Mild dementia	1				
Psychological problems	2				
Pressure sores or skin ulcers					
Yes	0				
No	1				
Dietary Assessment					
How many full meals does the patient eat daily?					
1 meal	0				
2 meals	1				
3 meals	2				
Consumes at least 1 serving of dairy products (milk, cheese, yogurt) per day					
No	1				
Yes	0				
Consumes 2 or more servings of Legumes or eggs per week					
No	0				
Yes	0.5				
Consumes meat, fish or poultry every day					
No	0				
Yes	1				
Consumes 2 or more servings of fruits or vegetables per day?					
No	0				
Yes	1				
Has food intake declined over the past 3 months due to loss of appetite?					
Severe loss of appetite	0				
Moderate loss of appetite	1				
No loss of appetite	2				
How much fluids consumed per day?					
< 5 glasses	0				
5–9 glasses	0.5				
> 9 glasses	1				
Mode of feeding					
With assistance	0				
Self-feed with some difficulty	1				
Self-feed without any problem	2				
Self Assessment					
Do they view themselves as having nutritional problems?					
Major malnutrition	0				
Does not know	1				
No nutritional problems	2				
In comparison with other people of same age, how they consider their health status?					
Not as good	0				
Does not know	0.5				
As good	1				
Better	2				
Max MNA score (best-nourished)	30				

Legend: The numbering on the left-hand side of score titles corresponds to the numbering of factors in Appendix 1

## Appendix 3

Illustrates how the Silver Code is calculated.

**Table 6:** Calculation of the Silver Code

Factor	Points
Age	
75–79	0
80–84	3
85+	9
Gender	
Female	0
Male	2
Marital status	
Married	0
Unmarried/widowed/divorced	1
Previous admission to a day hospital	
No	0
Yes	5
Previous admission to a regular ward and discharge diagnosis	
No admission (0)	0
Respiratory disease (6)	6
Cancer (11)	11
Other (2)	2
Number of drugs in the previous 3 mo	
0–8	0
8+	2
Total score: 0 pts. = best possible performance, 36 pts. = worst possible performance– Corresponds to gradient risk for mortality	

## Appendix 4

Describes the components of the Short Physical Performance Battery (SPPB).

**Table 7:** Short Physical Performance Battery (SPPB)

Instructions	Scoring
1. Repeated Chair Stands	
• Ask patient if they think it is safe for them to try and stand up from a chair five times without using their arms. • If yes, instruct patient to stand up straight and then sit back down again as quickly as they can five times, without stopping in between, keeping their arms folded over their chest. • Demonstrate. • Begin the stopwatch when patient begins to stand up. Count aloud each time patient rises. • Stop the stopwatch when subject has straightened up completely for the fifth time. Also stop if the subject uses arms, if they have not completed rises after 1 min, or if concerned about their safety.	- <5 stands completed in ≤1 min = 0 pts. - 5 stands in >16.7 s and ≤1 min = 1 pt. - 5 stands in 16.6–13.7 s = 2 pts. - 5 stands in 13.6–11.2 s = 3 pts. - 5 stands in <11.1 s = 4 pts
2. Balance Testing	
*Semitandem:* • Instruct patient to stand with the side of the heel of one foot touching the big toe of the other foot for 10 s (left/right feet as preferred by patient). • Demonstrate • Stand next to patient to help them into a semitandem position, allowing them to hold onto your arms to establish balance. • Begin timing when patient has the feet in position. • If *unable* to hold the semitandem position for 10 s → *side-by-side.* • If *able* to hold the semitandem position for 10 s *→ tandem.* → *Side-by-side*: As for semitandem but with feet together. Patients may use their arms, bend their knees, or move their body to maintain balance, but may not move their feet. → *Tandem*: As for semitandem but with the heel of one foot in-front-of and touching the toes of the other foot.	- Side-by-side: <10 s or unable = 0 pts. - Side-by-side: ≥10 s; semitandem: <10 s = 1 pt. - Semitandem: ≥10 s; tandem: 0–2 s = 2 pts. - Semitandem: ≥10 s; tandem: 3–9 s = 3 pts. - Tandem: ≥10 s = 4 pts
3. Eight-foot (2.44 m) walk	
• Instruct patient to walk at their usual pace to the other end of course (a distance of 8 ft) and to continue walking until they pass the end of the tape. If they use a cane or other walking aid outside of their home, they should use it for the test. • Press the start button on the stopwatch as the participant begins walking. Walk with the patient. • Measure the time they take to complete the 8-ft course.	- Unable to complete course = 0 pts. - Completed course in >5.7 s = 1 pt. - Completed course in 4.1–6.5 s = 2 pts. - Completed course in 3.2–4.0 s = 3 pts. - Completed course in <3.1 s = 4 pts
SPPB score: 0 pts. = worst possible performance, 12 pts. = best performance Score corresponds to gradient risk for mortality, nursing home admission, and disability	

Legend: *pt.* point, *SPPB* short physical performance battery

## Appendix 5

Details about procedural aspects are provided.

**Table 8:** Procedural characteristics

	n/N (%)
Access route	
Transfemoral	55/71 (77.5)
Transapical	9/71 (12.7)
Transaortic	7/71 (9.9)
Type of THV	
SAPIEN XT	26/71 (36.6)
SAPIEN 3	40/71 (56.3)
Other	5/71 (7.0)
THV diameter	
23 mm	37/70 (52.9)
26 mm	26/70 (37.1)
29 mm	7/70 (10.0)
Second valve used	2/71 (2.8)
Pre-implantation balloon dilatation	63/71 (88.7)
Post-delivery balloon dilatation	9/71 (12.7)

Legend: *THV* transcatheter heart valve

## Appendix 6

Details about periprocedural outcomes are provided.

**Table 9:** Periprocedural outcomes

	Mean ± SD / n/N (%)
Device success (VARC-2)^a^	
Absence of procedural mortality	70/70 (100.0)
Correct positioning of THV	70/70 (100.0)
Intended performance of THV	70/70 (100.0)
Intraoperative complications^b^	12/70 (17.1)
Vascular complications requiring treatment^c^	7/70 (10.0)
Access-related complications (dissection, rupture)	4/71 (5.6)
Conversion to open surgery (%)	0/70 (0.0)
Paravalvular regurgitation (%)	
None/trace	46/70 (65.7)
Mild	22/70 (31.4)
Moderate	2/70 (2.9)
Severe	0/70 (0.0)
Transvalvular leakage (%)	
None/trace	62/70 (88.6)
Mild	8/70 (11.4)
Moderate/severe	0/70 (0.0)
AV peak gradient (mmHg)	20.0 ± 12.4 (*n* = 31)
AV mean gradient (mmHg)	11.3 ± 7.2 (*n* = 32)

Legend: *THV* transcatheter heart valve, *AV* aortic valve

^a^Valve Academic Research Consortium criteria: absence of procedural mortality, correct positioning of a single prosthetic heart valve into proper anatomical position, and intended performance of the prosthetic heart valve (no prosthesis–patient mismatch) and mean aortic valve gradient

^b^Includes access-related complications (8 pts), asystole/arrhythmia (2 pts), haemorrhagic stroke (1 pt) [one patient no further information available]

^c^Includes aneurysm, haematoma, pericardial haematoma/effusion (2 pts), apical bleeding (1 pt)

## Appendix 7

Changes of the CGA over 3 month.

**Table 10:** CGA baseline vs 3 months

	Baseline		3 months		Intra-individual change			
	N	Mean ± SD	N	Mean ± SD	N	Mean ± SD	95% CI	*p*-value
MPI total score	71	0.34 ± 0.11	56	0.30 ± 0.13	56	−0.02 ± 0.12	−0.05, 0.01	0.25
ADL	71	0.05 ± 0.15	60	0.07 ± 0.22	60	0.04 ± 0.25	−0.02, 0.11	0.20
IADL	71	0.16 ± 0.29	60	0.21 ± 0.32	60	0.06 ± 0.32	−0.02, 0.14	0.16
SPMSQ	71	0.01 ± 0.06	57	0.02 ± 0.09	57	0.01 ± 0.12	−0.02, 0.04	0.57
CIRS	71	0.82 ± 0.27	56	0.71 ± 0.38	56	−0.12 ± 0.25	−0.18, −0.05	< 0.001
MNA	71	0.30 ± 0.33	56	0.21 ± 0.28	56	−0.07 ± 0.37	−0.17, 0.03	0.16
ESS	71	0.08 ± 0.22	56	0.02 ± 0.09	56	−0.04 ± 0.13	−0.07, 0.0	0.04
Medication use	71	0.86 ± 0.24	60	0.88 ± 0.21	60	0.04 ± 0.27	−0.03, 0.11	0.23
Co-habitation status	71	0.42 ± 0.50	60	0.31 ± 0.46	60	−0.08 ± 0.39	−0.18, 0.03	0.14
Silver code	71	22.53 ± 6.44	57	22.8 ± 6.33	57	−0.31 ± 3.55	−0.64, 1.25	0.52
SPPB	71	5.69 ± 3.33	56	7.82 ± 2.84	56	1.86 ± 2.76	1.12, 2.60	< 0.001

Legend: *MPI* Multidimensional Prognostic Index, *ADL* Activities of Daily Living, Instrumental Activities of Daily Living, *SPMSQ* Short Portable Mental Status Questionnaire, *CIRS* Cumulative Illness Rating Scale, *MNA* Mini Nutritional Assessment, *ESS* Exton-Smith Scale, *SPPB* short physical performance battery

## Abbreviations

ACC: American College of Cardiology
AS: Aortic stenosis
AV: Aortic valve
BMI: Body mass index
CGA: Comprehensive geriatric assessment
CHF: Congestive heart failure
CI: Confidence interval
CIRS: Cumulative illness rating scale
eCRF: Electronic case report form
ESS: Exton-Smith Scale
EUGMS: European union geriatric medicine society
IADL: Instrumental Activities of Daily Living
LVEF: Left ventricular ejection fraction
MACCCE: Major adverse cardiac or cerebrovascular event
MI: Myocardial infarction
MNA: Mini nutritional assessment
MPI: Multidimensional prognostic index
NYHA: New York Heart Association
OR: Odds ratio
SAVR: Surgical aortic valve replacement
SPMSQ: Short portable mental status questionnaire
SPPB: Short physical performance battery
STS: Society of thoracic surgeons
TAVI: Transcatheter aortic valve implantation
TIA: Transient ischaemic attack
TUG: Timed get-up and go
VARC: Valve Academic Research Consortium
